# Anatomic and neurochemical analysis of the palpal olfactory system in the red flour beetle *Tribolium castaneum*, HERBST

**DOI:** 10.3389/fncel.2023.1097462

**Published:** 2023-02-23

**Authors:** Björn Trebels, Stefan Dippel, Janet Anders, Clara Ernst, Brigitte Goetz, Tim Keyser, Karl Heinz Rexer, Ernst A. Wimmer, Joachim Schachtner

**Affiliations:** ^1^Animal Physiology, Department of Biology, Philipps-University Marburg, Marburg, Germany; ^2^Biodiversity of Plants, Department of Biology, Philipps-University Marburg, Marburg, Germany; ^3^Department of Developmental Biology, Johann-Friedrich-Blumenbach-Institute for Zoology and Anthropology, Georg-August-University Göttingen, Göttingen, Germany; ^4^Clausthal University of Technology, Clausthal-Zellerfeld, Germany

**Keywords:** *Tribolium castaneum*, insect, olfaction, chemosensation, neuroanatomy, gnathal olfactory center, lobus glomerulatus, palpal sensilla

## Abstract

The paired antennal lobes were long considered the sole primary processing centers of the olfactory pathway in holometabolous insects receiving input from the olfactory sensory neurons of the antennae and mouthparts. In hemimetabolous insects, however, olfactory cues of the antennae and palps are processed separately. For the holometabolous red flour beetle *Tribolium castaneum*, we could show that primary processing of the palpal and antennal olfactory input also occurs separately and at distinct neuronal centers. While the antennal olfactory sensory neurons project into the antennal lobes, those of the palps project into the paired glomerular lobes and the unpaired gnathal olfactory center. Here we provide an extended analysis of the palpal olfactory pathway by combining scanning electron micrographs with confocal imaging of immunohistochemical staining and reporter expression identifying chemosensory and odorant receptor-expressing neurons in the palpal sensilla. In addition, we extended the anatomical characterization of the gnathal olfactory center by 3D reconstructions and investigated the distribution of several neuromediators. The similarities in the neuromediator repertoire between antennal lobes, glomerular lobes, and gnathal olfactory center underline the role of the latter two as additional primary olfactory processing centers.

## 1 Introduction

To find hosts, conspecifics, mates, oviposition sites, and food sources, insects often depend on their chemical senses (Visser, 1986; Laska, 1999; Liu et al., 2008; Whiteman and Pierce, 2008; Yang et al., 2008; Dicke, 2009; Weiss et al., 2011; Stensmyr et al., 2012; Sun et al., 2012; Weissteiner et al., 2012; Linz et al., 2013; Paczkowski et al., 2014). The chemosensory systems are precisely tuned to discriminate between chemical cues. They translate perceived information based on composition, concentration, and spatial and temporal distribution, into innate and learned behavior.

Chemosensation starts with the perception of the semiochemicals at the chemosensory sensilla of the antennae and palps. The sensilla house the chemosensory neurons (CSNs) that divide into olfactory sensory neurons (OSNs) and gustatory sensory neurons. The OSNs present the olfactory receptors, either odorant receptors (ORs) or ionotropic glutamate-like receptors (IRs), on their membranes (Sato et al., 2008; Wicher et al., 2008; Benton et al., 2009; Missbach et al., 2014; Dippel et al., 2016). The OSNs relay the perceived olfactory information to their respective primary processing centers *via* axons. The antennal OSNs project into the antennal lobes (ALs), while the destination of the palpal OSNs differs between species. For the ALs, it is postulated that OSNs expressing the same specific OR converge onto a single glomerulus (Vosshall, 2000). In hemimetabolous insects, the palpal OSNs project into the glomerular lobes—also called lobus glomerulatus (LGs) (Ernst et al., 1977; Ignell et al., 2000; Schachtner et al., 2005), which are believed to be fused with the ALs in most well studied holometabolous insects (Anton and Homberg, 1999). Consequently, in holometabolous insects, the ALs are commonly declared as primary processing centers for antennal and palpal olfactory input (Anton and Homberg, 1999; Vosshall, 2000; Szyszka and Galizia, 2015; Lin et al., 2018).

Within the ALs, the olfactory information perceived by the OSNs is processed by a complex network of different neuron types comprised of local interneurons (LNs), projection neurons (PNs), and centrifugal neurons (Schachtner et al., 2005). The LNs interconnect the glomeruli and shape the olfactory representations mainly by the inhibitory transmitter gamma amino-butyric acid (GABA) or the excitatory transmitter acetylcholine (Stopfer et al., 1997; Sachse and Galizia, 2002; Wilson and Laurent, 2005; Olsen et al., 2007, 2010; Shang et al., 2007; Silbering and Galizia, 2007; Olsen and Wilson, 2008; Okada et al., 2009; Tanaka et al., 2009; Chou et al., 2010; Root, 2010; Wilson, 2013; Nagel et al., 2015). In addition, biogenic amines and a diverse set of neuropeptides further modulate the olfactory representation (Predel et al., 2004; Schachtner et al., 2005; Nässel and Homberg, 2006; Altstein and Nässel, 2010; Carlsson et al., 2010; Binzer et al., 2014; Siju et al., 2014). The processed information is then forwarded from the ALs by the PNs to higher brain centers: the mushroom bodies and the lateral horns (Schachtner et al., 2005; Galizia and Rössler, 2010; Dippel et al., 2016).

**Figure 1:**
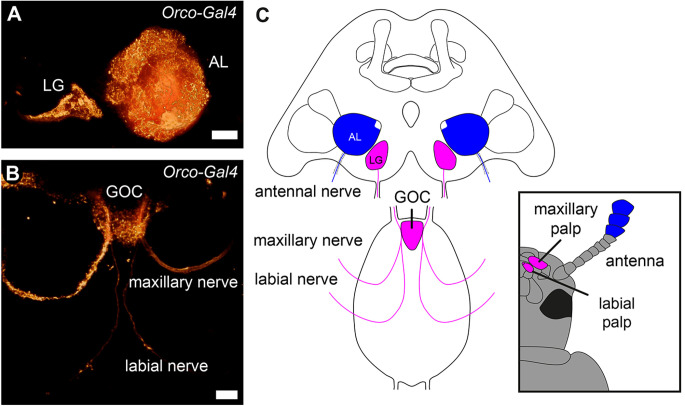
Primary olfactory pathways in the red flour beetle *Tribolium castaneum*. Volume rendering of the reporter signal in the OSN-labeling partial Orco-GAL4 line **(A)** of the glomerular lobes (LG), antennal lobes (AL), and **(B)** the gnathal olfactory center (GOC) with its sensory inputs. **(C)** Schematic of the primary processing centers and their sensory inputs. Antennal OSNs (blue) project into the ALs, while the palpal OSNs (magenta) project into the GOC and LGs. Scale bars 20 μm **(A)**, 10 μm **(B)**.

However, in mosquitoes, a suboesophageal zone receiving olfactory innervation from a gnathal appendage has been identified (Riabinina et al., 2016). Moreover, in the red flour beetle, the palpal OSNs do not project into the ALs but into the paired LGs and the unpaired, glomerularly organized gnathal olfactory center (GOC; Figure 1; Dippel et al., 2016). Here, we provide a detailed analysis, of whether in *T. castaneum* the GOC and LGs serve as additional primary olfactory processing centers besides the ALs. Thereby, we focus on an anatomical analysis of the palpal olfactory pathway and a neurochemical analysis of the GOC and LGs based on the transmitter repertoire described for the ALs in this beetle (Binzer et al., 2014; Trebels et al., 2021).

## 2 Material and methods

### 2.1 Animals

All experiments were performed using red flour beetles (*Tribolium castaneum*, HERBST 1797; Insecta, Coleoptera) of the wild-type strain “San Bernadino” (Sokoloff, 1966), the transgenic CSN-labeling EF1-B-DsRed line (elongation factor1-alpha regulatory region-DsRedExpress; kindly provided by Michalis Averof, Institut de Génomique Fonctionnelle de Lyon, France; Posnien et al., 2011; Dippel et al., 2016), or the OSN-labeling partial Orco-Gal4 line (Dippel et al., 2016). The beetles were bred under constant darkness at about 30°C (wildtype) or 28°C (transgenes) and 40%–50% relative humidity on organic whole grain wheat flour supplemented with 5% dried yeast powder and 0.05% Fumagilin-B (Medivet Pharmaceuticals Ltd., High River, Alberta, Canada) to prevent sporozoan infections (Berghammer et al., 1999). For age determination, freshly eclosed beetles (A0) were collected and kept in mixed-sex groups of 20 in 68 ml *Drosophila* vials on about 20 g substrate.

### 2.2 Histochemical staining

The brains and/or gnathal ganglia of cold anesthetized beetles (usually 3–5 per staining) of known age and sex were dissected in PBS (phosphate-buffered saline, 0.01 M, pH 7.4) and fixed in 0.01 M PBS containing 4% paraformaldehyde for 1–2 h at room temperature or 4°C overnight. Fixation was stopped by rinsing 4 × 10 min in PBS supplemented with 0.3% Triton X-100 (PBS-TrX). Blocking was performed in either 5% normal goat serum or normal donkey serum (both Jackson Immuno Research, Westgrove, PA, USA) for 3–4 h at room temperature or overnight at 4°C. The brains and/or gnathal ganglia were then incubated with the primary antibody solution (PBS-TrX, 2% normal serum). After 2–3 days at 4°C, the antibodies were removed by rinsing for 5 × 10 min in PBS-TrX. Subsequently, the brains and/or gnathal ganglia were incubated for 2–3 days at 4°C in constant darkness with secondary antisera and fluorescent markers in buffer solution (PBS-TrX, 2% normal serum). Concentrations of primary and secondary antibodies are listed in Table 1). Following staining, brains and/or gnathal ganglia were either mounted aqueous in Mowiol (Mowiol embedding medium, 2010) or after dehydration in an ascending ethanol series (50%, 70%, 90%, 95%, 100%, 100%; 3 min each) and cleared with methyl salicylate (Merck, Gernsheim, Germany) in Permount mounting medium (Fisher Scientific, Pittsburgh, PA) as a whole between two coverslips using a layer of two reinforcing rings as spacers to prevent squeezing.

**Table 1:** Overview of used antibodies and markers.

Name	Abbreviation	Host species	Dilution	Vendor/donor (Catalog #, Batch #, RRID #)	Reference	Specificity in T. castaneum
Drosophila melanogaster Synapsin I (SYNORF1)	Synapsin	Mouse	1:50	E. Buchner, University of Würzburg, Germany (n/a, n/a, AB_2313617)	Klagges et al. (1996)	Utz et al. (2008)
Locusta migratoria Tachykinin II	TKRP	Rabbit	1:20,000	Jena Bioscience, Jena, Germany (CLK-AZ118–1; Kli009-030; AB_2341129)	Veenstra et al. (1995)	Binzer et al. (2014)
Rattus norvegicus glutamate decarboxylase (sheep)	GADsheep	Sheep	1:5,000	W. Oertel, Laboratory of Clinical Science, Mansfield, MA, USA (n/a; n/a;n/a)	Oertel et al. (1981)	Trebels et al. (2021)
Rattus norvegicus glutamate decarboxylase (rabbit)	GADrabbit	Rabbit	1:1,000	Sigma-Aldrich; now Merck KGaA, Darmstadt, Germany (G5163; 113M4772; AB_477019)		Trebels et al. (2021)
Periplaneta americana myoinhibitory peptide I	MIP	Rabbit	1:5,000	M. Eckert, University of Jena, Germany (n/a, n/a, AB_2314803)	Predel et al. (2001)	Binzer et al. (2014)
Manduca sexta allatotropin	AT	Rabbit	1:5,000	J. Veenstra, University of Bordeaux, France (n/a, n/a, AB_2313973)	Veenstra and Hagedorn (1993)	Binzer et al. (2014)
5-Hydroxy -Tryptamine (serotonin)	5-HT	Rabbit	1:20,000	Immunostar, Hudson, WI, USA (20080; 924005; AB_572263)		
Red fluorescent protein	DsRed	Chicken	1:3,000	Rockland Immunochemicals INC, Limerick, PA, USA (600–901–379, 26274, AB_10704808)		
Moth-R2, Orco antiserum	Moth-R2	Rabbit	1:5,000	J. Krieger, University Halle-Wittenberg, Germany	Dippel et al. (2016)	Dippel et al. (2016)
Cy 3 coupled goat anti-chicken	GACh-Cy 3	Goat	1:300	Jackson ImmunoResearch; Westgrove, PA, USA (103-165-155, 93117, AB_2337386)		
Cy 3 coupled goat anti-rabbit	GAR-Cy 3	Goat	1:300	Jackson ImmunoResearch; Westgrove, PA, USA (111-165-144, n/a, AB_2338006)		
Cy 5 coupled goat anti-mouse	GAM-Cy 5	Goat	1:300	Jackson ImmunoResearch; Westgrove, PA, USA (115-175-146, n/a, AB_2338713)		
Cy 2 coupled donkey anti-sheep	DAS-Cy 2	Donkey	1:300	Jackson ImmunoResearch; Westgrove, PA, USA (713-225-147, n/a, AB_2340735)		
Alexa Fluor 488 coupled phalloidin	Phalloidin		1:200	Thermo Fischer Scientific, Rockford, IL, USA (A12379; n/a; n/a)	Vandekerckhove et al. (1985)	

### 2.3 Expansion microscopy (ExM)

We adapted the original protocol for intact, thick tissues (Asano et al., 2018). All buffers and solutions were prepared following the original protocol. In short, following the incubation with the secondary antisera, the ganglia were equilibrated in 2-(N-morpholino) ethanesulfonic acid buffered saline (MBS) for 30 min and rinsed twice for 15 min each in MBS. Afterward, ganglia were treated according to the original protocol. The ganglia were incubated in 1:100 Acryloyl-X /DMSO in MBS for 16–24 h at room temperature before rinsing twice in PBS for 15 min. This was followed by the addition of an acrylamide gelling solution in which the ganglia were incubated for 45–60 min at 4°C. The ganglia and the gelling solution were transferred into a gelation chamber (here build from a microscope slide and coverslips) and incubated for 2 h at 37°C to allow for polymerization of the gel. The polymerized gel was then trimmed to the dimensions of the ganglia and incubated for 24 h at room temperature in the digestion buffer containing Proteinase K. This was followed by quickly rinsing three times for 20 min in pure water at 4°C which started the expansion which is completed after. Afterward, specimens were embedded in a mixture of glycerol and PBS (80% glycerol, 20% PBS) between two coverslips using a layer of two reinforcing rings as spacers.

### 2.4 Image acquisition and analysis

Fluorescent preparations including preparations obtained after ExM were imaged using a confocal laser scanning microscope (TCS SP2 or TCS SP5, Leica Microsystems, Wetzlar, Germany). Overviews were taken at 10x magnification, while detail images were taken at either 40x or 63x magnification. Digitization was generally performed at 1,024 × 1,024 or 2,048 × 2,048 pixel resolution, a pinhole-size of 1 airy, a line average of 2–4, and a z-step size between 0.5 and 2.5 μm. The resulting image stacks were analyzed with Amira graphics software (FEI SAS a part of Thermo Fisher Scientific, Mérignac Cedex, France) and images were exported in TIF format. Further image processing (global level adjustments, contrast, and brightness optimization) was performed in Photoshop CC (Adobe Systems, San Jose, CA, USA). Final figure arrangements were made in Illustrator CC (Adobe Systems). Videos were edited using Final Cut Pro (Apple Inc. Cupertino, CA, USA).

### 2.5 Scanning electron microscopy

Cold anesthetized adult beetles were decapitated, and the heads were fixed overnight in 4% formaldehyde in 0.01 M PBS at 4°C and afterward washed in PBS. Washed samples were then dehydrated in an ascending acetone series (30%, 50%, 3 × 100%). Finally, acetone was allowed to evaporate overnight. After sputtering with gold (Balzers Union Sputter Coater, Balzers, Lichtenstein; Quorum Technologies Ltd, Ringmer, UK), specimens were examined using an SEM (S-530, Hitachi High-Technologies Europe GmbH, Krefeld, Germany). Micrographs were taken using a digital image acquisition unit (DISS 5, point electronic, Halle, Germany). Further image processing (global level adjustments, contrast, and brightness optimization) was performed in Photoshop CC (Adobe Systems, San Jose, CA, USA). Final figure arrangements were made in Illustrator CC (Adobe Systems).

## 3 Results

### 3.1 OSNs innervate the sensilla of labial and maxillary palpal tips

As previously shown, the labial and maxillary palps of *T. castaneum* are olfactory sensory appendages, whose sensory input is processed in the paired LGs in the cerebral ganglion and/or the unpaired GOC in the gnathal ganglion (Dippel et al., 2016; Figure 1).

**Figure 2:**
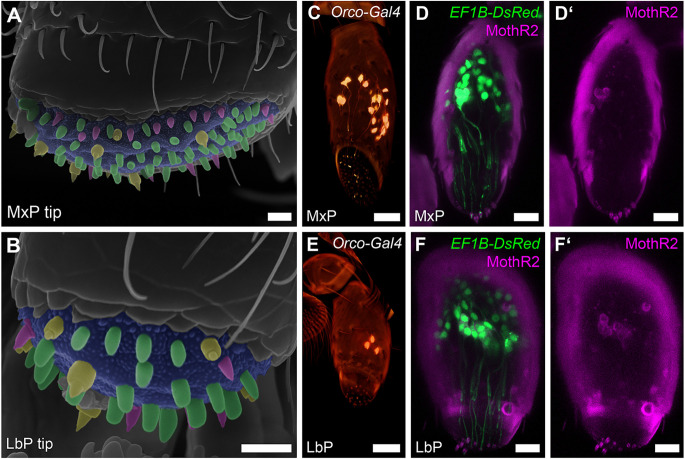
OSNs of the maxillary and labial palps. **(A,B)** Scanning electron micrographs of the tips of the maxillary (MxP) and labial (LbP) palps and their sensilla: basiconic sensilla (sBas)—magenta, blunt basiconic sensilla (sBBas)—green, and styloconic sensilla (sSty)—yellow, Scale bars 5 μm. **(C,E)** Volume rendering of the reporter signal in the Orco-GAL4 × UAS-DsRed line in maxillary palps (MxP) and labial palps (LbP), Scale bars 20 μm. **(D,D’,F,F’)** Orco immunostaining (MothR2; magenta) and reporter signal (green) on palpal cryosections in the CSN-labeling EF-1-B-DsRed line within the maxillary (MxP) and labial palps (LbP). The cuticle is visible in the same channel due to autofluorescence, Scale bars 10 μm.

To identify, which palpal sensilla are innervated by the previously described OSNs, whose somas are exclusively located in the last segment of the maxillary and labial palps (Dippel et al., 2016), we first determined the sensilla types at the palpal tips using scanning electron microscopy (SEM). On both, the maxillary and labial palps (Figures 2A,B), we found the same three types of sensilla and classified them according to Roth and Willis (1951) as basiconic sensilla (magenta), blunt basiconic sensilla (green), and styloconic sensilla (yellow). Based on exemplary manual counting in SEM images, we estimate that the tip of the maxillary palp houses roughly 90 sensilla divided into about 20 (*n* = 2; #1: 22, #2: 16) basiconic, 60 (*n* = 2; #1: 63, #2: 57) blunt basiconic, and 10 (*n* = 2; #1: 13, #2: 8) styloconic sensilla. The labial palp’s tip houses roughly 32 sensilla divided into about 11 (*n* = 2; #1: 12, #2: 11) basiconic, 15 (*n* = 2; #1: 19, #2: 12) blunt basiconic, and 6 (*n* = 2; #1: 6, #2: 7) styloconic sensilla. Secondly, we analyzed the OSN dendrites marked by a partial Orco-GAL4 line and by specific antibody staining against Orco in the CSN labeling EF-1-B-DsRed line and could show that they innervate these sensilla at the palpal tips (Figures 2C,D,D’,E,F,F’). By high-resolution confocal imaging, we identified that the styloconic (sSty; Supplemental Figures S1A,A’), the basiconic (sBas), and/or blunt basiconic (sBBas) sensilla house these OSN dendrites (Supplemental Figures S1B,B’). A differentiation between basiconic and blunt basiconic sensilla in the confocal images was not possible due to resolution limits in imaging the cuticular autofluorescence.

### 3.2 Glomerular organization of the GOC

**Figure 3:**
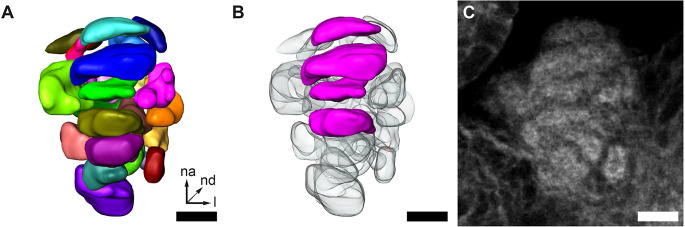
GOC anatomy. **(A,B)** Representative 3D reconstruction of the GOC. **(B)** Wedge-like glomeruli (magenta) in the same GOC as shown in **(A)**. These are recognizable across specimens depicted. **(C)** Single optical slice of the phalloidin staining of the GOC reconstructed in **(A)** and **(B)**. All scale bars 10 μm. The orientation given in **(A)** applies to all panels—na, n-anterior; nd, n-dorsal; l, lateral.

To further elucidate the glomerular organization of the GOC, we performed manual 3D-reconstructions (Figure 3A) based on the fluorescent reporter signal in the EF-1-B-DsRed line and fluorescent staining of f-actin and synapsin (Figure 3C). On average, we identified about 30 glomeruli (mean = 30.33, SD = 4.78, *n* = 9) of varying shape and size, which was confirmed by two exemplary 3D reconstructions after applying expansion microscopy (ExM), which improves spatial resolution by uniformly expanding the tissue within a gel matrix (Asano et al., 2018). In both cases, one female and one male, 31 glomeruli were identified. We usually find about four stacked and repeatedly identifiable wedge-like glomeruli in comparable positions (Figure 3B), while the other glomeruli could not be exactly correlated between specimens.

### 3.3 Local neurons of the GOC and LGs

The vast majority of AL LNs use GABA as the major inhibitory transmitter. Therefore, we investigated whether GABA is also used in the GOC and LGs by immunocytochemistry against glutamic acid decarboxylase (GAD), the enzyme synthesizing GABA.

**Figure 4:**
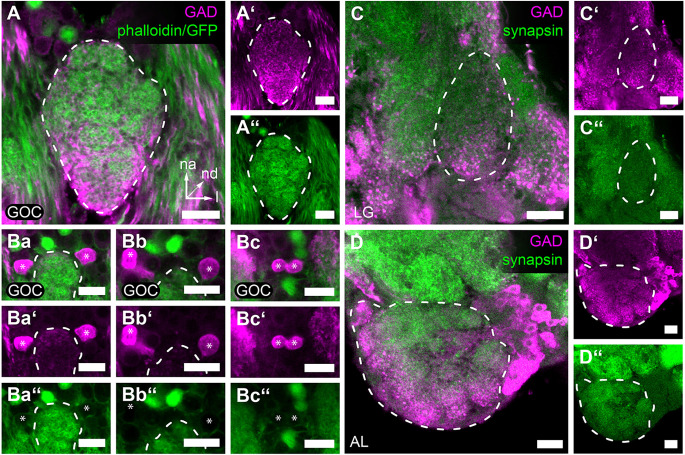
GAD immunoreactivity in the primary olfactory processing centers. Representative optical sections of GAD immunostaining (magenta) in the **(A,A’,Ba–Bc,Ba’–Bc’)** GOC, **(C,C’)** LG, and **(D,D’)** AL. **(A,A’)** GAD-immunoreactive fibers cover the complete GOC. **(Ba,Ba’,Bb,Bb’,Bc,Bc’)** GAD-immunoreactive soma (asterisks) n-anterior near the GOC. Some **(Ba,Ba’,Bb,Bb’)** extend fibers into the GOC volume (see Supplementary Video). **(C,C’)** GAD-immunoreactive fibers are distributed within the whole volume of the LG, while somas could not be detected in the near vicinity. **(D,D’)** In the AL, GAD immunoreactive somas locate lateral to the glomeruli and enter the neuropile *via* a single tract. In **(A–C,A’–C’)** the staining was obtained using the GAD_rabbit_ antisera and in **(D,D’)** using the GAD_sheep_ antisera. The general neuroanatomy is depicted in green. In **(A,A”,Ba–Bc,Ba”–Bc”)** *via* phalloidin staining and 3XP3-eGFP reporter signal in the EF-1-B-DsRed line and in **(C,C”,D,D”)** *via* synapsin immunostaining. Dashed lines indicate neuropil outlines. All scale bars 10 μm. Orientation given in **(A)** applies to all panels—na, n-anterior; nd, n-dorsal; l, lateral.

In the GOC, we find GAD immunoreactive fibers that penetrate the glomeruli throughout the neuropil volume (Figures 4A–A”). We identified six nearby GAD immunoreactive somas n-anterior to the GOC (Figures 4Ba–Ba”,Bb–Bb”,Bc–Bc” marked with asterisks), of which four (*n* = 2) were confirmed to extend their neurites into the GOC neuropil (Figures 4Bb–Bb”,Bc–Bc”). This is similar to the situation in the ALs (Trebels et al., 2021), where GAD immunostaining is visible in all glomeruli and a cluster of cell bodies locate lateral to the AL neuropil (Figures 4D–D”).

The LGs also display GAD immunoreactivity, however, no corresponding cell bodies with neurites extending into the LGs could be identified in the near vicinity (Figures 4C–C”).

### 3.4 Neuropeptide co-transmitters in the GOC and LGs

Based on the known neuropeptide repertoire and distribution in the red flour beetles ALs (Binzer et al., 2014), we investigated the distribution of allatotropin (AT), tachykinin-related peptides (TKRP), and myoinhibitory peptides (MIPs) in the GOC and the LGs, and compared it to the AL.

**Figure 5:**
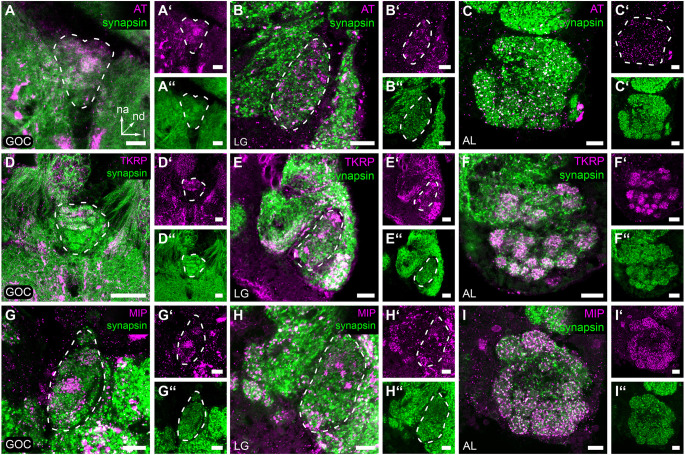
Neuropeptides in the primary olfactory processing centers. Representative optical slices displaying neuropeptide immunostaining (magenta) in the gnathal olfactory center (GOC), the glomerular lobes (LG), and the antennal lobes (AL). **(A–C,A’–C’)** Allatotropin (AT) immunostaining shows a dense pattern in the GOC **(A,A’)**, while the pattern a in the LGs **(B,B’)** and AL **(C,C’)** shows a more speckly distribution. **(D–F,D’–F’)** Immunostaining of Tachykinin related peptides (TKRP) reveals identifiable glomeruli in the n-anterior portion GOC **(D,D’)**, dense spots, likely resembling microglomeruli in the LGs **(E,E’)**, and a glomerular pattern in the complete AL **(F,F’)**. **(G–I,G’–I’)** Immunostaining against Myoinhibitory peptides (MIP) visualizes only a few glomeruli of the GOC **(G,G’)**, dense spots across the complete LG **(H,H’)**, and identifiable glomeruli in the total AL **(I,I’)**. **(A–I,A”–I”)** The general neuroanatomy (green) is visualized *via* synapsin immunostaining. Dashed lines indicate neuropil outlines. All scale bars 10 μm. The orientation given in **(A)** applies to all panels—na, n-anterior; nd, n-dorsal; l, lateral.

Immunohistochemical staining of AT in the GOC revealed a dense pattern with visible glomerular substructures in its n-anterior part (Figures 5A–A”). In contrast, immunostaining of AT in LGs revealed a speckled pattern (Figures 5B–B”) similar to the AL (Figures 5C–C”). The staining covers the total neuropil volume of the ALs and LGs. In the GOC, besides some glomeruli at the n-posterior end, all including the wedge-like glomeruli are labeled.

In the GOC, the TKRP-immunostaining (Figures 5D–D”) reveals a glomerular pattern similar to that found in the AL (Figures 5F–F”). The n-anterior two-thirds of the GOC including the wedge-like glomeruli are labeled (Figure 5D”). In contrast, in the LGs dense immunoreactive spots are detected all over the neuropil volume (Figures 5E–E”).

MIPs are found only in a few glomeruli of the GOC (Figures 5G–G”), which is in contrast to the broad distribution in the ALs (Figures 5I–I”). In the LGs (Figures 5H–H”), dense spots are detected all over the neuropil volume.

### 3.5 Potential modulation of odor detection by serotonin in the GOC, LGs, and ALs

**Figure 6:**
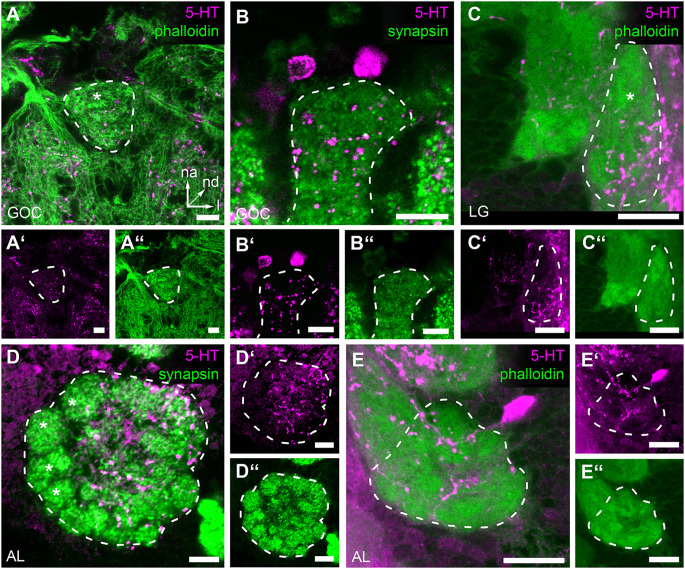
Serotonin (5-HT) immunoreactivity in the primary olfactory processing centers. Representative optical slices displaying Serotonin (5-HT) immunostaining in the antennal lobes (AL), glomerular lobes (LG), and the gnathal olfactory center (GOC) **(A,A’)** The GOC is only partially innervated by immunoreactive fibers; the asterisk indicates the position of non-innervated glomeruli. **(B,B’)** Serotonin immunoreactive neurons that likely innervate the GOC. **(C,C’)** In the LGs immunoreactive fibers only cover a part of the neuropil; the asterisk indicates the position of the non-innervated area. **(D,D’)** In the ALs immunoreactive fibers cover most but not all glomeruli; the asterisks indicate non-innervated glomeruli. **(E,E’)** Single Serotonin-immunoreactive neuron innervating the ipsilateral AL. The general neuroanatomy (green) is either depicted *via* phalloidin immunostaining **(A,A”,C,C”,E,E”)** or staining synapsin **(B,B”,D,D”)**. Dashed lines indicate neuropil outlines. All scale bars 10 μm. Orientation given in **(A)** applies to all panels—na, n-anterior; nd, n-dorsal; l, lateral.

To evaluate the possibility that olfactory responses in the GOC, LGs, and ALs are modulated by serotonin (5-HT) immunoreactivity against this biogenic amine was analyzed. The immunostaining of the GOC revealed that most of the glomeruli are encapsulated by a mesh of serotonin immunoreactive fibers (Figures 6A–A”)—except for a few n-anterior located glomeruli including the four wedge-like glomeruli (Figure 6A asterisk). The sources of the serotonergic fibers are very likely two cells, whose somas locate in the n-anterior cell cluster (Figures 6B–B”) and whose neurites extend towards the GOC.

In the LGs, 5-HT immunoreactivity was limited to the lateral n-posterior portion of the neuropil, where varicose ramifications are observed (Figures 6C–C”). The corresponding somas, however, could not be identified.

The soma of a serotonergic neuron locates lateral to each AL and extends fibers into the ipsilateral AL (Figures 6D–D”,E–E”). Within the ALs, these fibers encapsulate most but a few glomeruli located n-anterolateral (Figure 6D, asterisks) with varicose ramifications.

## 4 Discussion

In the red flour beetle *T. castaneum*, besides the antennae, also the maxillary and labial palps are major olfactory sensory organs (Dippel et al., 2016). While the antennal olfactory pathway and the transmitter repertoire of the antennal lobe are well described (Dreyer et al., 2010; Binzer et al., 2014; Dippel et al., 2016; Trebels et al., 2021), only basic anatomical data has been available on the palpal olfactory pathway (Dippel et al., 2016), for which this study provides now a more detailed picture. This includes the morphology of sensilla types on the palpal tips and whether they are innervated by OSNs, the glomerular organization of the GOC, potential local neurons of the GOC, and a first insight into the neuro-mediator repertoire of the GOC and LGs.

### 4.1 The palps as olfactory sensory organs

The maxillary and labial palps of *T. castaneum* both house a reasonable number of OSNs and thus are major olfactory appendages (Dippel et al., 2016). The cell bodies of the OSNs are located near the base of the sensilla within the distal segment of the maxillary and labial palps, while their dendrites extend into the sensilla shaft. Based on SEM of the maxillary and labial palps, we confirmed the same three sensilla types as previously described (Roth and Willis, 1951): basiconic, blunt basiconic, and styloconic sensilla. Therefore the palps of *T. castaneum* employ multiple olfactory sensilla types, while the maxillary palps of* D. melanogaster* harbor only basiconic sensilla (Bruyne et al., 1999).

In contrast to the antennae, the palps harbor styloconic but not coeloconic sensilla. Since the coeloconic sensilla are possible hosts for ionotropic glutamate-like receptor-expressing neurons (Dippel et al., 2016), and at least the IR-Co receptors (IR25a and IR76b) are significantly expressed in *T. castaneum’s* mouthparts (Dippel et al., 2016) it is thus possible that the styloconic sensilla on the palp are housing both IR or OR expressing OSNs. The expressed GRs (Dippel et al., 2016) are most likely housed in gustatory neurons of basiconic sensilla, as this has been postulated for the antenna in *T. castaneum* (Dippel et al., 2016) and sugar response has been shown in the sister species *T. breviconis* (Alabi et al., 2014) for antennal basiconic sensilla.

The semiochemicals detected by the palpal OSNs of *T. castaneum* remain unknown. However, given the exposed position of the sensilla, those OSNs are most likely involved in short-range attraction such as the palpal OSNs in flies (Dweck et al., 2016), or food evaluation such as the proboscis OSNs in *Manduca sexta* (Haverkamp et al., 2016). Moreover, in the locust, *Locusta migratoria*, in which palpation behavior preceding food uptake is observed (Blaney and Duckett, 1975), the palpal OSNs project into the LGs and mitigate vomiting behavior and thus play a role in food evaluation and rejection (Sun et al., 2022).

### 4.2 Anatomy of the GOC and LGs

The 3D reconstructions of the red flour beetle’s GOC confirmed with about 30 glomeruli the previous rough estimation (Dippel et al., 2016). The recognition of four clearly identifiable wedge-like glomeruli in the GOC suggests at least two important analogies to the glomerular organization of the ALs. First, glomeruli that differ in size and/or form may serve special tasks, like the enlarged glomeruli of males that have been described in a variety of insects and that are presumably involved in the processing of pheromone signaling (for review see Schachtner et al., 2005). Secondly, a glomerular organization with individual glomeruli positioned at similar sites within ALs of different individuals follows a concept in olfactory systems organization that has been deduced from *D. melanogaster* (Jefferis, 2005; Smith, 2009). Our finding supports the hypothesis that GOC and AL follow the same logic to process odor information.

The microglomerular organization of the paired LGs differs from the GOC and ALs. However, microglomerular organization of olfactory processing centers has been found in the ALs of the caeliferans *Schistocerca gregaria* and *Chorthippus albomarginatus* (Ignell et al., 2001), some beetles such as *Acilius sulcatus* and *Ilybius fuliginosus* (Panov, 2014; Kollmann et al., 2016), as well as in the mushroom body calyces of various insects, including the scarab beetle *Eudicella woermanni* (Groh and Rössler, 2011).

### 4.3 Local neurons of the GOC and LGs

GABA has been described as the major inhibitory transmitter of AL LNs in diverse insects (Hoskins et al., 1986; Schäfer and Bicker, 1986; Distler, 1990; Leitch and Laurent, 1996; Seidel and Bicker, 1997; Wegerhoff, 1999; Schachtner et al., 2005; Okada et al., 2009) and the distribution of GAD immunoreactivity in the ALs of *T. castaneum* (Trebels et al., 2021; Figures 4D–D”) resembles the findings of GABA immunoreactivity in other hemi- and holometabolous insect species (Hoskins et al., 1986; Schäfer and Bicker, 1986; Distler, 1990; Leitch and Laurent, 1996; Wegerhoff, 1999). LGs and GOC showed innervation by GAD immunoreactive fibers throughout the neuropils (Figures 4A–A”,C–C”), indicating that in both neuropils inhibitory LNs modulate odor processing. For the GOC, GAD- immunoreactive cell bodies in close vicinity (Figures 4Ba–Ba”,Bb–Bb”) are likely the source of the GAD-immunoreactive fibers (Figures 4Bc–Bc”). In the case of the LGs, we could not identify the origin of the innervating GAD- immunoreactive fibers. Their innervation probably stems from LNs shared with the AL, which is believed to be fused with LGs in many other holometabolous insects (Anton and Homberg, 1999).

### 4.4 Neuropeptide co-transmitters in the GOC and LGs

**Figure 7:**
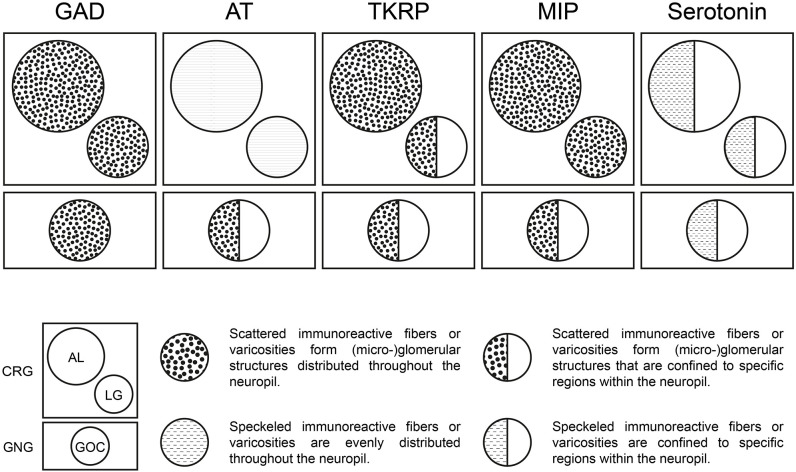
Neuromediators in the primary olfactory processing centers of *T. castaneum*. Distribution patterns of GAD, AT, TKRP, MIP, and Serotonin within the ALs, LGs, and GOC.

Neuropeptides are primarily considered to be co-transmitters at synaptic sites (Nässel and Zandawala, 2019). In vertebrates, they are mainly believed to function as neuromodulators (Nässel and Zandawala, 2019). In the ALs of *T. castaneum* and many other insect species neuropeptides are predominantly localized in LNs and the number of mass spectrometry-identified neuropeptides ranges between 20 and 40 (Carlsson et al., 2010; Binzer et al., 2014; Siju et al., 2014). These include AT, TKRPs, and MIPs. The detection of all three peptides in LG and GOC suggests a strong similarity to the neuropeptide repertoire of and role in the ALs (Binzer et al., 2014). However, compared to the AL, the distribution pattern of immunostaining differs in GOC and LG (Figure 7). In contrast to the ALs, in which all three peptides are distributed throughout the neuropil, neuropeptide activity seems to be spatially restricted in the GOC and at least for TKRP in the LGs. Interestingly, serotonin shows spatial restriction in all three neuropils (see Section “4.5 Potential modulation of odor detection by serotonin in the GOC, LGs, and ALs”). LGs have so far not been described in other holometabolous insect species and are supposedly fused with the ALs (Anton and Homberg, 1999). For hemimetabolous insects, only limited data concerning neuropeptides in the LGs exists. As in the red flour beetle, AT immunoreactivity is distributed over the total volume of the LGs in *Schistocerca gregaria* (Homberg et al., 2004) and MIP-immunoreactivity (Schulze et al., 2012) as well as TKRP-immunoreactivity (Muren et al., 1995) are present in LGs of the madeira cockroach *Rhyparobia maderae*.

In general, only a few studies on the role of neuropeptides in olfactory signaling are available. For example, small neuropeptide F (sNPF) in the ALs is involved in increasing food-searching behavior in hungry *D. melanogaster* (Root, 2010), and in combination with AST-A reduced host-seeking behavior in *Aedes aegypti* (Christ et al., 2017). MIPs in the AL are involved in the same behavior in mated female flies (Hussain et al., 2016). Further, studies showed that TKRPs in the ALs can modify odor sensitivity (Winther et al., 2006; Winther and Ignell, 2010) and seemingly play a role in regulating food attraction (Ko et al., 2015). Given the potential role of MIPs and TRKPs in food-searching behavior, it is not surprising to find them in the primary processing center for palpal olfaction, with palpal OSNs likely being involved in short-range attraction and food evaluation.

### 4.5 Potential modulation of odor detection by serotonin in the GOC, LGs, and ALs

The serotonergic innervation of the GOC (Figures 6A–A”) is presumably provided *via* two serotonin-immunoreactive cell bodies in the near vicinity (Figures 6B–B”). For the LGs, we could not identify a serotonin-positive cell body as a source of the innervation (Figures 6C–C”). As for GABA, we suggest that the serotonin-immunoreactive fibers in the LG stem from AL-associated neurons. The serotonergic fibers innervating each AL of *T. castaneum* (Figures 6D–D”) arise from a single neuron located lateral to the respective AL (Figures 6E–E”). These neurons share their gross morphology with specific serotonin-immunoreactive neurons described in various other insect species, while their arborization patterns show high variability (Kent et al., 1987; Wegerhoff, 1999; Schachtner et al., 2005; Dacks et al., 2006; Coates et al., 2017). In the ALs of *T. castaneum*, the innervation pattern resembles a meshwork of varicosities distributed over the boundaries of most, but not all, glomeruli. Since not all glomeruli of the GOC and ALs are covered by serotonin-immunoreactive varicosities, serotonin-modulated odor processing seems to be restricted to specific glomeruli or happens outside the glomeruli (Zhang and Gaudry, 2016). This might also apply to the AL glomeruli in *D. melanogaster* (Singh et al., 2013; Coates et al., 2017). In general, serotonin is assumed to play a modulatory function in various contexts (Erber et al., 1993; Mercer et al., 1995, 1996), and in the AL, serotonin immunoreactive fibers form a conserved pattern in diverse insect species (Schürmann and Klemm, 1984; Tyrer et al., 1984; Kent et al., 1987; Rehder et al., 1987; Nässel, 1988; Strambi et al., 1989; Breidbach, 1990; Salecker and Distler, 1990). The role of serotonin in the olfactory system seems to be heterogeneous but is generally thought to modulate odor-evoked responsiveness and/or sensitivity (Lizbinski and Dacks, 2018): e.g., serotonin was shown to increase the responsiveness to olfactory signals in *M. sexta* (Kloppenburg et al., 1999; Dacks et al., 2008; Kloppenburg and Mercer, 2008) and *D. melanogaster* (Dacks et al., 2009; Zhang and Gaudry, 2016). Therefore, serotonin in the palpal olfactory system might elevate state-dependent responsiveness to olfactory cues involved in palpation and food evaluation.

## 5 Conclusion

The current study provides a more detailed anatomic analysis of the palpal olfactory system and gives a first insight into the neuromediator repertoire of the LGs and the GOC in holometabolous insects. Comparison of the glomerular organized GOC with the well-examined ALs showed many similarities, including individually identifiable glomeruli and similar neuromediator innervation, suggesting an important role of LGs and GOC in olfactory processing in addition to the ALs. The four individually identifiable wedge-like glomeruli are promising targets for future physiological and genetic experiments to provide insight into the role of a separate palpal olfactory system.

## Data availability statement

The original contributions presented in the study are included in the article/Supplementary material, further inquiries can be directed to the corresponding author/s.

## Author contributions

BT conceived and designed the study, acquired, analyzed, and interpreted the data; and drafted and revised the article. JA, CE, BG, TK, and KR acquired and analyzed the data. SD, EW, and JS conceived and designed the study, analyzed and interpreted the data, and drafted and revised the article. All authors contributed to the article and approved the submitted version.
