# Supplementary material for: Anatomic and neurochemical analysis of the palpal olfactory system in the red flour beetle Tribolium castaneum, HERBST
Source: Front Cell Neurosci. 2023 Feb 23;17:1097462. doi: 10.3389/fncel.2023.1097462 (PMC10043995; doi:10.3389/fncel.2023.1097462)
Supplement: Supplementary file 1 [file Data_Sheet_1.pdf]

Supplementary Material

1 Supplementary Figures

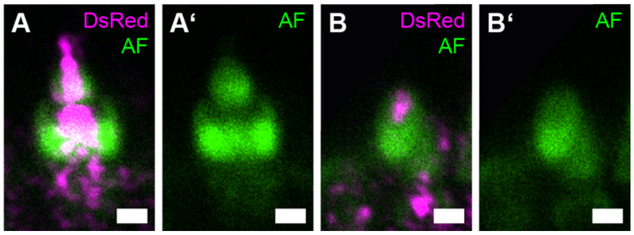

Fig. S1: Olfactory sensilla of the palps.
Optical slices from confocal image stacks of the DsRed reporter signal and cuticular autofluorescence (AF) in the Orco-Gal4xUAS-DsRed line showing A-A' styloconic sensilla and B-B' basiconic/blunt basiconic sensilla (differentiation not possible due to resolution limits in imaging cuticular autofluorescence). All scale bars 2µm.

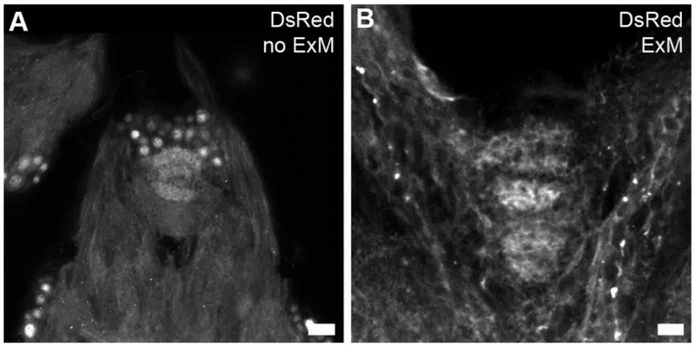

Fig. S2: Comparison of an unexpanded and an expanded GOC.
A Single optical slice of the n-dorsal part of a representative GOC as depicted by the DsRed reporter signal in the neuron labeling EF-1-B-DsRed line. B Single optical slice of the n-dorsal part of a representative GOC as depicted by the DsRed reporter signal in the neuron labeling EF-1-B-DsRed line after application of the ExM protocol. Scale bars 10µm.

2 Supplementary Videos

Supporting Videos to Figures 4, 5, and 6:
https://doi.org/10.5281/zenodo.7586511
